# 3-(Adamantan-1-yl)-4-(prop-2-en-1-yl)-1*H*-1,2,4-triazole-5(4*H*)-thione

**DOI:** 10.1107/S1600536812005065

**Published:** 2012-02-10

**Authors:** Maha S. Almutairi, Mona M. Al-Shehri, Ali A. El-Emam, Seik Weng Ng, Edward R. T. Tiekink

**Affiliations:** a Department of Pharmaceutical Chemistry, College of Pharmacy, King Saud University, Riyadh 11451, Saudi Arabia; b Department of Chemistry, University of Malaya, 50603 Kuala Lumpur, Malaysia; c Chemistry Department, Faculty of Science, King Abdulaziz University, PO Box 80203 Jeddah, Saudi Arabia

## Abstract

The title mol­ecule, C_15_H_21_N_3_S, exists as the thione tautomer in the solid state. The 1,2,4-triazole ring is almost planar (r.m.s. deviation = 0.004 Å) and the prop-2-en-1-yl chain is close to being perpendicular to this plane [C—N—C—C torsion angle = 77.1 (5)°]. In the crystal, centrosymmetric dimeric aggregates are formed by pairs of N—H⋯S hydrogen bonds as parts of eight-membered (⋯HNCS)_2_ synthons. These are connected into layers parallel to (101) *via* C—H⋯π inter­actions, where the π-system is the triazole ring. The investigated sample was a nonmerohedral twin; the refined domain ratio was 0.655 (4):0.345 (4).

## Related literature

For the biological activity of adamantyl derivatives, see: Vernier *et al.* (1969 ▶); El-Emam *et al.* (2004 ▶). Kadi *et al.* (2007 ▶, 2010 ▶). For the biological activity of adamantyl-1,2,4-triazole derivatives, see: Al-Deeb *et al.* (2006 ▶). For the separation of diffraction data into twin domains, see: Spek (2009 ▶).
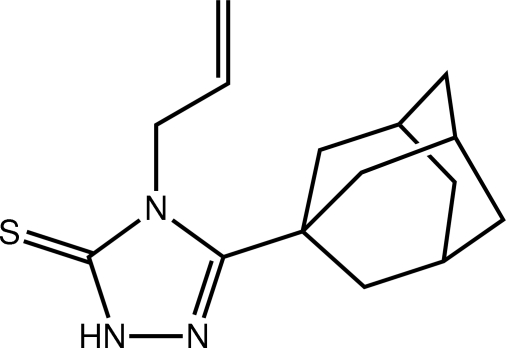


## Experimental

### 

#### Crystal data

C_15_H_21_N_3_S
*M* *_r_* = 275.41
Monoclinic, 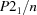

*a* = 13.5833 (17) Å
*b* = 8.6483 (6) Å
*c* = 13.6973 (14) Å
β = 115.938 (14)°
*V* = 1447.0 (3) Å^3^
*Z* = 4
Mo *K*α radiation
μ = 0.22 mm^−1^
*T* = 100 K
0.35 × 0.15 × 0.10 mm

#### Data collection

Agilent SuperNova Dual diffractometer with Atlas detector
Absorption correction: multi-scan (*CrysAlis PRO*; Agilent, 2011 ▶) *T*_min_ = 0.929, *T*_max_ = 0.979
10998 measured reflections
3324 independent reflections
2875 reflections with *I* > 2σ(*I*)
*R*_int_ = 0.077

#### Refinement

*R*[*F* ^2^ > 2σ(*F* ^2^)] = 0.081
*wR*(*F* ^2^) = 0.230
*S* = 1.17
3324 reflections
173 parameters
H-atom parameters constrained
Δρ_max_ = 0.71 e Å^−3^
Δρ_min_ = −0.66 e Å^−3^

### 

Data collection: *CrysAlis PRO* (Agilent, 2011 ▶); cell refinement: *CrysAlis PRO*; data reduction: *CrysAlis PRO*; program(s) used to solve structure: *SHELXS97* (Sheldrick, 2008 ▶); program(s) used to refine structure: *SHELXL97* (Sheldrick, 2008 ▶); molecular graphics: *ORTEP-3* (Farrugia, 1997 ▶) and *DIAMOND* (Brandenburg, 2006 ▶); software used to prepare material for publication: *publCIF* (Westrip, 2010 ▶).

## Supplementary Material

Crystal structure: contains datablock(s) global, I. DOI: 10.1107/S1600536812005065/hb6626sup1.cif

Structure factors: contains datablock(s) I. DOI: 10.1107/S1600536812005065/hb6626Isup2.hkl

Supplementary material file. DOI: 10.1107/S1600536812005065/hb6626Isup3.cml

Additional supplementary materials:  crystallographic information; 3D view; checkCIF report

## Figures and Tables

**Table 1:** Hydrogen-bond geometry (Å, °) *Cg*1 is the centroid of the C1/C2/N1/N2/N3 ring.

*D*—H⋯*A*	*D*—H	H⋯*A*	*D*⋯*A*	*D*—H⋯*A*
N2—H2N⋯S1^i^	0.88	2.43	3.296 (3)	170
C5—H5⋯*Cg*1^ii^	1.00	2.60	3.529 (6)	155
C13—H13*A*⋯*Cg*1^iii^	0.99	2.81	3.351 (5)	115

Symmetry codes: (i) 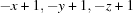; (ii) 
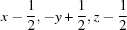
; (iii) 
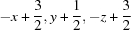
.

**Fig. 1.**
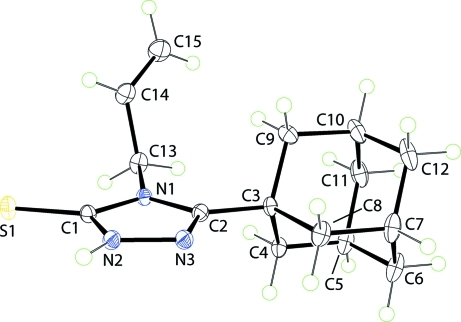
The molecular structure of (I) showing displacement ellipsoids at the 50% probability level.

**Fig. 2.**
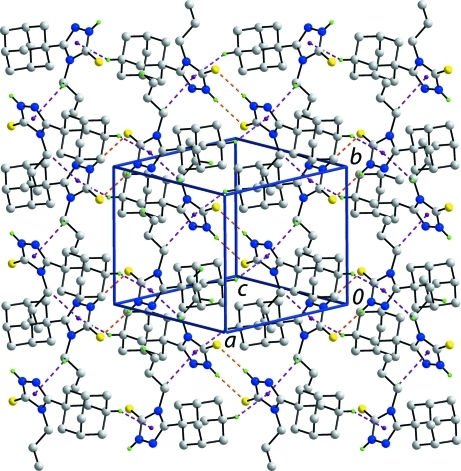
A view of the supramolecular layer parallel to (101) in (I). The N—H···S hydrogen bonds and C—H···π interactions are shown as orange and purple dashed lines, respectively.

**Fig. 3.**
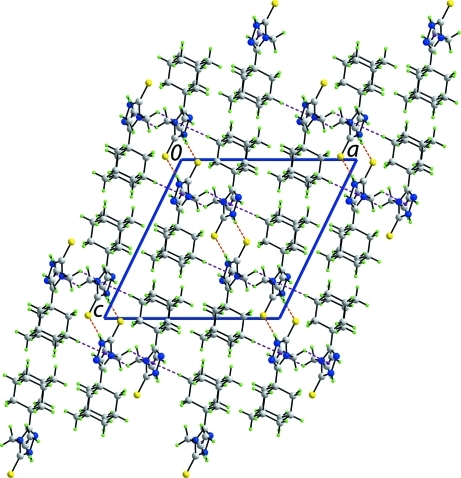
A view in projection down the a axis of the unit-cell contents for (I). The N—H···S, C—H···S and C—H···π interactions are shown as orange, blue and purple dashed lines, respectively.

## supplementary crystallographic information

### Comment

Derivatives of adamantane have long been known for their diverse biological activities including anti-viral activity against the influenza (Vernier *et al.*, 1969) and HIV viruses (El-Emam *et al.*, 2004). Moreover, adamantane derivatives were recently reported to exhibit marked anti-bacterial activity (Kadi *et al.*, 2007, 2010). In an earlier publication, we reported the synthesis and potent anti-microbial and anti-inflammatory activities for a series of 5-(1-adamantyl)-4-substituted-4*H*-1,2,4-triazole-3-thiols and related derivatives, including the title compound, (I) (Al-Deeb *et al.*, 2006). Herein, the crystal and molecular structure is described which was performed to determine the tautomeric form of (I).

The key result of the structure determination of (I) is the confirmation of the thione form of the molecule, Fig. 1. The 1,2,4-triazole ring is planar [r.m.s. deviation = 0.004 Å] and the S1 atom lies 0.060 (1) Å out of this plane. The C13 atom lies even further out of the plane, *i.e.* by 0.155 (4) Å in the opposite direction to the S1 atom. The prop-2-en-1-yl chain is almost perpendicular to the plane through the five-membered ring as seen in the value of the C1—N1—C13—C14 torsion angle of 77.1 (5)°. The terminal ethene bond is directed toward the adamantyl group.

In the crystal packing, centrosymmetric dimeric aggregates are formed by N—H···S hydrogen bonds *via* eight-membered {···HNCS}_2_ synthons. These are connected into a two-dimensional array parallel to (101) *via* C—H···π interactions, where the π-system is the triazole ring, Fig. 2 and Table 1. Layers stack without specific intermolecular interactions between them, Fig. 3.

### Experimental

A mixture of adamantane-1-carbohydrazide (1.94 g, 0.01 mol) and allyl isothiocyanate (0.99 g, 0.01 mol), in ethanol (10 ml) was heated under reflux with stirring for one hour and the solvent was distilled off *in vacuo*. Aqueous sodium hydroxide (10%, 15 ml) was added to the residue and the mixture was heated under reflux for 2 h then filtered hot. On cooling, the mixture was acidified with hydrochloric acid and the precipitated crude product was filtered, washed with water, dried and crystallized from aqueous ethanol to yield 2.18 g (79%) of (I) as colourless prisms. m.p. 468–470 K. ^1^H NMR (CDCl_3_): δ 1.75–1.83 (m, 6H, adamantane-H), 1.94 (s, 3H, adamantane-H), 2.05 (s, 6H, adamantane-H), 4.91 (s, 2H, CH_2_), 5.03 (d, 1H, ═CHa, J = 17.0 Hz), 5.30 (d, 1H, ═CHb, J = 10.5 Hz), 5.90–5.96 (m, 1H, –CH═), 11.78 (br s, 1H, NH). ^13^C NMR: δ 27.88, 35.52, 36.27, 38.58 (adamantane-C), 47.66 (CH_2_), 117.92 (═CH_2_), 131.03 (–CH═), 158.34 (C═N), 168.61 (C═S).

### Refinement

Carbon-bound H atoms were placed in calculated positions [N—H = 0.88 Å and C—H = 0.95 to 1.00 Å, *U*_iso_(H) = 1.2*U*_eq_(N, C)] and were included in the refinement in the riding model approximation.

A sphere of reflections was measured, which should be sufficient to refine the non-merohedral twinned structure. However, separating the reflection data into two domains did not lead to an improvement in the refinement, and this was not improved at varying degrees of overlap. The twin domains were instead separated by using the *TwinRotMat* routine of *PLATON* (Spek, 2009). The minor twin component refined to 34.5 (4)%.

Two reflections, *i.e.* (10 3 5) and (5 0 1), were omitted owing to poor agreement.

### Crystal data

**Table:**

C_15_H_21_N_3_S	*F*(000) = 592
*M**_r_* = 275.41	*D*_x_ = 1.264 Mg m^−^^3^
Monoclinic, *P*2_1_/*n*	Mo *K*α radiation, λ = 0.71073 Å
Hall symbol: -P 2yn	Cell parameters from 1371 reflections
*a* = 13.5833 (17) Å	θ = 2.4–27.5°
*b* = 8.6483 (6) Å	µ = 0.22 mm^−^^1^
*c* = 13.6973 (14) Å	*T* = 100 K
β = 115.938 (14)°	Prism, colourless
*V* = 1447.0 (3) Å^3^	0.35 × 0.15 × 0.10 mm
*Z* = 4	

### Data collection

**Table:**

Agilent SuperNova Dual diffractometer with Atlas detector	3324 independent reflections
Radiation source: SuperNova (Mo) X-ray Source	2875 reflections with *I* > 2σ(*I*)
Mirror monochromator	*R*_int_ = 0.077
Detector resolution: 10.4041 pixels mm^-1^	θ_max_ = 27.6°, θ_min_ = 2.8°
ω scans	*h* = −17→15
Absorption correction: multi-scan (*CrysAlis PRO*; Agilent, 2011)	*k* = −11→11
*T*_min_ = 0.929, *T*_max_ = 0.979	*l* = −6→17
10998 measured reflections	

### Refinement

**Table:**

Refinement on *F*^2^	Primary atom site location: structure-invariant direct methods
Least-squares matrix: full	Secondary atom site location: difference Fourier map
*R*[*F*^2^ > 2σ(*F*^2^)] = 0.081	Hydrogen site location: inferred from neighbouring sites
*wR*(*F*^2^) = 0.230	H-atom parameters constrained
*S* = 1.17	*w* = 1/[σ^2^(*F*_o_^2^) + (0.0882*P*)^2^ + 4.0546*P*] where *P* = (*F*_o_^2^ + 2*F*_c_^2^)/3
3324 reflections	(Δ/σ)_max_ < 0.001
173 parameters	Δρ_max_ = 0.71 e Å^−^^3^
0 restraints	Δρ_min_ = −0.66 e Å^−^^3^

### Special details

**Table:**

Geometry. All s.u.'s (except the s.u. in the dihedral angle between two l.s. planes) are estimated using the full covariance matrix. The cell s.u.'s are taken into account individually in the estimation of s.u.'s in distances, angles and torsion angles; correlations between s.u.'s in cell parameters are only used when they are defined by crystal symmetry. An approximate (isotropic) treatment of cell s.u.'s is used for estimating s.u.'s involving l.s. planes.
Refinement. Refinement of *F*^2^ against ALL reflections. The weighted *R*-factor *wR* and goodness of fit *S* are based on *F*^2^, conventional *R*-factors *R* are based on *F*, with *F* set to zero for negative *F*^2^. The threshold expression of *F*^2^ > 2σ(*F*^2^) is used only for calculating *R*-factors(gt) *etc*. and is not relevant to the choice of reflections for refinement. *R*-factors based on *F*^2^ are statistically about twice as large as those based on *F*, and *R*-factors based on ALL data will be even larger.

### Fractional atomic coordinates and isotropic or equivalent isotropic displacement parameters (Å^2^)

**Table:**

	*x*	*y*	*z*	*U*_iso_*/*U*_eq_	
S1	0.60052 (9)	0.72236 (11)	0.51878 (8)	0.0170 (3)	
N1	0.6426 (3)	0.7440 (4)	0.7336 (3)	0.0115 (6)	
N2	0.5548 (3)	0.5391 (4)	0.6557 (3)	0.0137 (7)	
H2N	0.5203	0.4685	0.6062	0.016*	
N3	0.5707 (3)	0.5286 (4)	0.7620 (3)	0.0141 (7)	
C1	0.5976 (3)	0.6683 (5)	0.6357 (3)	0.0127 (7)	
C2	0.6240 (3)	0.6536 (4)	0.8078 (3)	0.0124 (7)	
C3	0.6643 (3)	0.6857 (5)	0.9271 (3)	0.0124 (7)	
C4	0.7912 (3)	0.6936 (5)	0.9857 (3)	0.0152 (8)	
H4A	0.8224	0.5954	0.9747	0.018*	
H4B	0.8173	0.7784	0.9544	0.018*	
C5	0.8298 (4)	0.7216 (5)	1.1068 (3)	0.0190 (9)	
H5	0.9115	0.7286	1.1435	0.023*	
C6	0.7923 (4)	0.5877 (6)	1.1559 (3)	0.0238 (10)	
H6A	0.8176	0.6052	1.2346	0.029*	
H6B	0.8245	0.4895	1.1462	0.029*	
C7	0.6670 (4)	0.5770 (6)	1.0993 (3)	0.0222 (9)	
H7	0.6429	0.4885	1.1307	0.027*	
C8	0.6281 (3)	0.5500 (5)	0.9779 (3)	0.0176 (8)	
H8A	0.5473	0.5411	0.9419	0.021*	
H8B	0.6592	0.4520	0.9665	0.021*	
C9	0.6144 (3)	0.8368 (5)	0.9470 (3)	0.0170 (8)	
H9A	0.6361	0.9254	0.9149	0.020*	
H9B	0.5335	0.8296	0.9113	0.020*	
C10	0.6547 (4)	0.8630 (6)	1.0692 (3)	0.0228 (10)	
H10	0.6233	0.9618	1.0813	0.027*	
C11	0.7803 (4)	0.8738 (6)	1.1234 (3)	0.0232 (9)	
H11A	0.8069	0.8941	1.2020	0.028*	
H11B	0.8037	0.9605	1.0914	0.028*	
C12	0.6173 (4)	0.7278 (6)	1.1179 (4)	0.0263 (11)	
H12A	0.5364	0.7208	1.0826	0.032*	
H12B	0.6419	0.7447	1.1966	0.032*	
C13	0.6875 (3)	0.9016 (5)	0.7431 (3)	0.0157 (8)	
H13A	0.7316	0.9079	0.7017	0.019*	
H13B	0.7365	0.9237	0.8202	0.019*	
C14	0.5979 (4)	1.0210 (5)	0.7008 (3)	0.0180 (8)	
H14	0.5409	1.0080	0.6295	0.022*	
C15	0.5939 (4)	1.1433 (5)	0.7573 (4)	0.0234 (10)	
H15A	0.6497	1.1593	0.8288	0.035*	
H15B	0.5353	1.2149	0.7264	0.035*	

### Atomic displacement parameters (Å^2^)

**Table:**

	*U*^11^	*U*^22^	*U*^33^	*U*^12^	*U*^13^	*U*^23^
S1	0.0220 (6)	0.0180 (5)	0.0114 (5)	−0.0044 (4)	0.0077 (4)	−0.0016 (4)
N1	0.0121 (15)	0.0121 (14)	0.0112 (15)	−0.0009 (13)	0.0058 (12)	0.0001 (12)
N2	0.0165 (16)	0.0116 (15)	0.0109 (14)	−0.0005 (13)	0.0039 (12)	−0.0013 (12)
N3	0.0173 (16)	0.0125 (16)	0.0114 (15)	0.0001 (13)	0.0051 (12)	−0.0014 (12)
C1	0.0131 (17)	0.0137 (18)	0.0104 (16)	0.0001 (15)	0.0043 (13)	−0.0016 (14)
C2	0.0137 (18)	0.0119 (17)	0.0127 (17)	−0.0005 (14)	0.0067 (14)	0.0010 (14)
C3	0.0135 (18)	0.0150 (18)	0.0108 (16)	−0.0018 (15)	0.0073 (14)	0.0003 (14)
C4	0.0156 (19)	0.0188 (19)	0.0121 (17)	−0.0012 (16)	0.0070 (14)	0.0014 (15)
C5	0.0162 (19)	0.025 (2)	0.0129 (18)	−0.0087 (17)	0.0038 (15)	0.0021 (16)
C6	0.023 (2)	0.031 (2)	0.0132 (18)	−0.0073 (19)	0.0042 (16)	0.0063 (17)
C7	0.023 (2)	0.029 (2)	0.0146 (18)	−0.0090 (19)	0.0088 (16)	0.0029 (17)
C8	0.0174 (19)	0.019 (2)	0.0155 (18)	−0.0065 (16)	0.0063 (15)	0.0016 (15)
C9	0.0181 (19)	0.0167 (19)	0.0173 (19)	−0.0002 (16)	0.0087 (16)	−0.0039 (15)
C10	0.026 (2)	0.030 (2)	0.0175 (19)	−0.0006 (19)	0.0137 (17)	−0.0061 (17)
C11	0.027 (2)	0.029 (2)	0.0150 (18)	−0.0116 (19)	0.0107 (17)	−0.0096 (17)
C12	0.024 (2)	0.043 (3)	0.016 (2)	−0.009 (2)	0.0134 (17)	−0.0073 (19)
C13	0.0161 (19)	0.015 (2)	0.0144 (17)	−0.0059 (15)	0.0054 (15)	−0.0007 (14)
C14	0.021 (2)	0.0132 (18)	0.0178 (18)	−0.0031 (16)	0.0071 (16)	0.0015 (15)
C15	0.030 (2)	0.015 (2)	0.024 (2)	−0.0007 (18)	0.0103 (18)	0.0003 (17)

### Geometric parameters (Å, º)

**Table:**

S1—C1	1.685 (4)	C7—C12	1.539 (7)
N1—C1	1.372 (5)	C7—H7	1.0000
N1—C2	1.390 (5)	C8—H8A	0.9900
N1—C13	1.476 (5)	C8—H8B	0.9900
N2—C1	1.342 (5)	C9—C10	1.534 (6)
N2—N3	1.379 (5)	C9—H9A	0.9900
N2—H2N	0.8800	C9—H9B	0.9900
N3—C2	1.300 (5)	C10—C11	1.537 (6)
C2—C3	1.504 (5)	C10—C12	1.538 (7)
C3—C8	1.550 (5)	C10—H10	1.0000
C3—C9	1.551 (6)	C11—H11A	0.9900
C3—C4	1.552 (5)	C11—H11B	0.9900
C4—C5	1.525 (5)	C12—H12A	0.9900
C4—H4A	0.9900	C12—H12B	0.9900
C4—H4B	0.9900	C13—C14	1.506 (6)
C5—C6	1.533 (6)	C13—H13A	0.9900
C5—C11	1.539 (7)	C13—H13B	0.9900
C5—H5	1.0000	C14—C15	1.326 (6)
C6—C7	1.534 (6)	C14—H14	0.9500
C6—H6A	0.9900	C15—H15A	0.9500
C6—H6B	0.9900	C15—H15B	0.9500
C7—C8	1.527 (6)		
			
C1—N1—C2	107.5 (3)	C7—C8—C3	110.3 (3)
C1—N1—C13	121.1 (3)	C7—C8—H8A	109.6
C2—N1—C13	131.0 (3)	C3—C8—H8A	109.6
C1—N2—N3	112.9 (3)	C7—C8—H8B	109.6
C1—N2—H2N	123.6	C3—C8—H8B	109.6
N3—N2—H2N	123.6	H8A—C8—H8B	108.1
C2—N3—N2	104.6 (3)	C10—C9—C3	110.0 (3)
N2—C1—N1	104.2 (3)	C10—C9—H9A	109.7
N2—C1—S1	128.1 (3)	C3—C9—H9A	109.7
N1—C1—S1	127.7 (3)	C10—C9—H9B	109.7
N3—C2—N1	110.8 (3)	C3—C9—H9B	109.7
N3—C2—C3	122.6 (3)	H9A—C9—H9B	108.2
N1—C2—C3	126.4 (3)	C9—C10—C11	109.1 (3)
C2—C3—C8	108.2 (3)	C9—C10—C12	109.4 (4)
C2—C3—C9	111.5 (3)	C11—C10—C12	110.1 (4)
C8—C3—C9	108.1 (3)	C9—C10—H10	109.4
C2—C3—C4	111.4 (3)	C11—C10—H10	109.4
C8—C3—C4	107.5 (3)	C12—C10—H10	109.4
C9—C3—C4	110.1 (3)	C10—C11—C5	110.0 (4)
C5—C4—C3	110.2 (3)	C10—C11—H11A	109.7
C5—C4—H4A	109.6	C5—C11—H11A	109.7
C3—C4—H4A	109.6	C10—C11—H11B	109.7
C5—C4—H4B	109.6	C5—C11—H11B	109.7
C3—C4—H4B	109.6	H11A—C11—H11B	108.2
H4A—C4—H4B	108.1	C10—C12—C7	108.7 (4)
C4—C5—C6	109.6 (3)	C10—C12—H12A	110.0
C4—C5—C11	109.4 (3)	C7—C12—H12A	110.0
C6—C5—C11	109.3 (4)	C10—C12—H12B	110.0
C4—C5—H5	109.5	C7—C12—H12B	110.0
C6—C5—H5	109.5	H12A—C12—H12B	108.3
C11—C5—H5	109.5	N1—C13—C14	111.4 (3)
C5—C6—C7	109.4 (4)	N1—C13—H13A	109.3
C5—C6—H6A	109.8	C14—C13—H13A	109.3
C7—C6—H6A	109.8	N1—C13—H13B	109.3
C5—C6—H6B	109.8	C14—C13—H13B	109.3
C7—C6—H6B	109.8	H13A—C13—H13B	108.0
H6A—C6—H6B	108.2	C15—C14—C13	123.7 (4)
C8—C7—C6	109.7 (4)	C15—C14—H14	118.2
C8—C7—C12	110.0 (4)	C13—C14—H14	118.2
C6—C7—C12	109.6 (4)	C14—C15—H15A	120.0
C8—C7—H7	109.2	C14—C15—H15B	120.0
C6—C7—H7	109.2	H15A—C15—H15B	120.0
C12—C7—H7	109.2		
			
C1—N2—N3—C2	0.0 (4)	C11—C5—C6—C7	60.0 (5)
N3—N2—C1—N1	−0.1 (4)	C5—C6—C7—C8	59.5 (5)
N3—N2—C1—S1	177.5 (3)	C5—C6—C7—C12	−61.4 (5)
C2—N1—C1—N2	0.2 (4)	C6—C7—C8—C3	−60.4 (5)
C13—N1—C1—N2	−173.0 (3)	C12—C7—C8—C3	60.3 (5)
C2—N1—C1—S1	−177.5 (3)	C2—C3—C8—C7	−179.9 (3)
C13—N1—C1—S1	9.3 (6)	C9—C3—C8—C7	−59.1 (4)
N2—N3—C2—N1	0.1 (4)	C4—C3—C8—C7	59.7 (4)
N2—N3—C2—C3	−176.5 (3)	C2—C3—C9—C10	178.3 (3)
C1—N1—C2—N3	−0.2 (5)	C8—C3—C9—C10	59.6 (4)
C13—N1—C2—N3	172.1 (4)	C4—C3—C9—C10	−57.5 (4)
C1—N1—C2—C3	176.2 (4)	C3—C9—C10—C11	59.2 (5)
C13—N1—C2—C3	−11.5 (7)	C3—C9—C10—C12	−61.3 (5)
N3—C2—C3—C8	−1.5 (5)	C9—C10—C11—C5	−61.2 (5)
N1—C2—C3—C8	−177.5 (4)	C12—C10—C11—C5	58.9 (4)
N3—C2—C3—C9	−120.2 (4)	C4—C5—C11—C10	61.1 (4)
N1—C2—C3—C9	63.8 (5)	C6—C5—C11—C10	−58.9 (4)
N3—C2—C3—C4	116.4 (4)	C9—C10—C12—C7	60.6 (5)
N1—C2—C3—C4	−59.6 (5)	C11—C10—C12—C7	−59.3 (4)
C2—C3—C4—C5	−178.4 (3)	C8—C7—C12—C10	−60.2 (4)
C8—C3—C4—C5	−60.1 (4)	C6—C7—C12—C10	60.6 (4)
C9—C3—C4—C5	57.4 (4)	C1—N1—C13—C14	77.1 (5)
C3—C4—C5—C6	61.0 (5)	C2—N1—C13—C14	−94.3 (5)
C3—C4—C5—C11	−58.8 (4)	N1—C13—C14—C15	126.8 (4)
C4—C5—C6—C7	−59.8 (5)		

### Hydrogen-bond geometry (Å, º)

Cg1 is the centroid of the C1/C2/N1/N2/N3 ring.

**Table:**

*D*—H···*A*	*D*—H	H···*A*	*D*···*A*	*D*—H···*A*
N2—H2*N*···S1^i^	0.88	2.43	3.296 (3)	170
C5—H5···*Cg*1^ii^	1.00	2.60	3.529 (6)	155
C13—H13*A*···*Cg*1^iii^	0.99	2.81	3.351 (5)	115

Symmetry codes: (i) −*x*+1, −*y*+1, −*z*+1; (ii) *x*−1/2, −*y*+1/2, *z*−1/2; (iii) −*x*+3/2, *y*+1/2, −*z*+3/2.
